# Amyotrophic lateral sclerosis in Beijing: Epidemiologic features and prognosis from 2010 to 2015

**DOI:** 10.1002/brb3.1131

**Published:** 2018-10-19

**Authors:** Shenghan Zhou, Yuliang Zhou, Silin Qian, Wenbing Chang, Liping Wang, Dongsheng Fan

**Affiliations:** ^1^ Beijing Advanced Innovation Center for Big Data‐Based Precision Medicine, School of Reliability and Systems Engineering Beihang University Beijing China; ^2^ Department of Neurology Peking University Third Hospital Beijing China

**Keywords:** amyotrophic lateral sclerosis, epidemiology, incidence, motor neuron diseases, prognosis

## Abstract

**Objective:**

To determine the incidence of amyotrophic lateral sclerosis (ALS) in Beijing from 2010 to 2015 and to address the issue of prognosis.

**Methods:**

The number of patients diagnosed with ALS was generated from two aspects, namely, diagnostic hospitals and assisted care institutions. By examining the consistency of the overlapping data in terms of age and gender distributions, the number of ALS patients in Beijing was estimated to analyze the incidence. Finally, a prognosis study was carried out by sorting the clinical data of deceased patients to associate time to death with the demographic characteristics, including gender, age at diagnosis, site of onset, body mass index, and lag from onset to diagnosis.

**Results:**

The average yearly incidence was 0.8/100,000 persons, the male–female ratio was 1.63:1, and the mean age at diagnosis was 54.11 years. The mean time from symptom onset to diagnosis was 14.8 months, and the median survival time from diagnosis was 49.4 months. In addition, each of the identified clinical features was related to the survival of the patients with ALS.

**Conclusions:**

The incidence of ALS in Beijing is similar to the rates in Hong Kong and Taiwan but is lower than the rates in Europe and America. In addition, the mean age at onset of the patients in Beijing was early, and overall ALS prognosis appears to be comparable to those reported in recent publications.

## 1 INTRODUCTION

Amyotrophic lateral sclerosis (ALS), also known as motor neuron disease (MND), is a progressive neurodegenerative disease involving the major motor neurons of the cerebral cortex, brain stem, and spinal cord (Kiernan et al., 2011). The disease is associated with age and is characterized by a poor prognosis and high mortality. The highest incidence has been observed between 55  and 75 year olds. Worldwide, most patients die within 2–4 years of the diagnosis of ALS, and only a few patients survive for at least 10 years. Little is known about the pathogenesis of ALS; approximately 5%–10% of ALS cases can be attributed to familial inheritance, while the rest may be related to environmental factors (Valle et al., 2015).

Most epidemiological data on ALS are derived from Europe, where population‐based registry studies have found great variability in the incidence of this disease, ranging from 0.3 to 2.8/100,000 persons‐years, according to the period, study area, ethnicity, or methodology. While the incidence of ALS in North America is similar to that in Europe, significant differences have been reported around the world, from 0.6 per 100,000 in Japan to 2.8 per 100,000 in Australia (Marin et al., 2016).

Epidemiologic studies of rare diseases are critical. However, to date, no studies of the incidence of ALS in mainland China have been conducted. Therefore, this study aimed to assess the ALS incidence and survival in a cohort of Beijing patients.

## 2 MATERIALS AND METHODS

### 2.1 Materials

The data were from two sources: the patient visit record of the Peking University Third Hospital (PUTH) and the census report of the Beijing Oriental Rain ALS care center (BORALS). PUTH is the main ALS diagnostic hospital in Beijing, where patients who are diagnosed with ALS choose a care center for adjuvant therapy, for example, the BORALS.

By deleting information regarding non‐Beijing patients from the census report, we obtained data from 283 patients between 2010 and 2015 in the BORALS. In addition, we defined this population as the first cohort (Table 1).

**Table 1:** The basic composition and content of the cohort

Cohort	Data Sources	Number of cases	Male	Female	M‐F	Included information
First cohort	BORALS	283	179	104	1.72	Demographic data (including residence, occupation, and lifestyle habits.), clinical data (time of onset, site of onset, time of diagnosis, diagnostic hospital, treatment, and basic body condition, etc.) The patients of the BORALS came from different hospitals: PUTH, The General Hospital of the People's Liberation Army (PLAGH), Peking Union Medical College Hospital (PUMCH), Beijing Tian Tan Hospital (BJTTH), Xuanwu Hospital Capital Medical University (XWHOSP), Beijing Boren Hospital (BBH), The General Hospital of the Navy PLA (NPLAGH), The General Hospital of Chinese people's Armed Police Force (CAPFGH). Among them, a few patients came from BBH, NPLAGH, and CAPFGH, which are represented by “other.”
Second cohort	PUTH	562	346	216	1.60	Patient name, gender, age, date of diagnosis, diagnosis result, residence, and other basic outpatient information
Third cohort	Merge and delete overlap	680	421	259	1.63	The intersection of the above two datasets, and the same as the second cohort

Note

M‐F: Male‐to‐female ratio.

The patient visit record of PUTH showed patient diagnostic records from 2010 to 2015. We identified 562 patients who were first diagnosed with ALS in PUTH during the prior 6 years, and this group was defined as the second cohort (Table 1).

By merging the above two datasets and deleting the overlap from the same patients, we obtained a more complete cohort. The new cohort consisted of 680 Beijing patients with ALS and was defined as the third cohort (Table 1).

The contents of the three cohorts were not all the same, as shown in Table 1. Furthermore, only patients fulfilling the El Escorial revised diagnostic criteria (Brooks, Miller, Swash, & Munsat, 2000) for definite, probable or probable laboratory‐supported and possible ALS and residing in the city of Beijing at the time of diagnosis of the disease were included in the present study.

### 2.2 Methods

**Figure 1:**
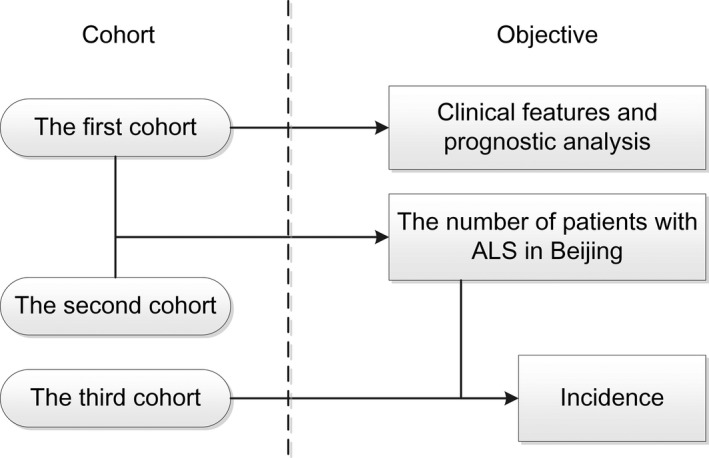
Study roadmap

According to the different information contained in the cohorts, we carried out different studies (see Figure 1). As expected, there was overlap between patients of the BORALS and PUTH, and we used this characteristic to estimate the number of patients in Beijing, as discussed below. The age distribution and sex ratio of all patients with ALS in Beijing were defined according to the third cohort because the number of cases in the third cohort was more complete than those in the other cohorts.

### 2.3 Estimation of the number of patients with ALS in Beijing

First, we present the hypothesis that the BORALS, as an assisted care institution, received ALS patients in Beijing and treated them as random. Moreover, patients were randomized at the BORALS after being diagnosed at each hospital in Beijing. In other words, the patients in the first cohort were a subset of all patients with ALS in Beijing under the case of random sampling. The distribution of each diagnostic hospital in the BORALS represents the proportion of all Beijing patients with ALS in each diagnostic hospital. To prove the above hypothesis, this paper tested the similarity and consistency of several important demographic data. Here, the demographic data were based on the age distribution and sex ratio of overlapping patients. If the hypothesis is confirmed, the total number of patients in Beijing could be estimated by using the two source datasets. The specific calculation process was as follows:

$$\frac{\alpha }{M}=\frac{\beta_{X}}{N_{X}}$$

where α is the proportion of patients from PUTH in the first cohort, and *M* is the total number of patients diagnosed with ALS in PUTH during the 6‐year period. Here, *M* = 562; $\beta_{X}$is the proportion of patients from X hospital in the first cohort; *N_X_* is the total number of patients diagnosed by X hospital in the first cohort; and X is the hospital described in Table 1.

The total number of patients with ALS in Beijing during the six‐year period was the sum of all *N_X_*.

### 2.4 Statistical methods

Microsoft EXCEL 2010 was used for data input and cleaning. SPSS version 19 was used for statistical description and the related calculation analyses. Empirical data distribution fit and goodness of fit tests were performed with the Anderson–Darling test (A–D) using Minitab version 16. Comparisons were performed with the chi‐square test and Kaplan–Meier survival analysis, with *p* values <0.05 considered statistically significant (Traxinger, Kelly, Johnson, Lyles, & Glass, 2013).

## 3 RESULTS

### 3.1 The number of patients with ALS in Beijing

**Figure 2:**
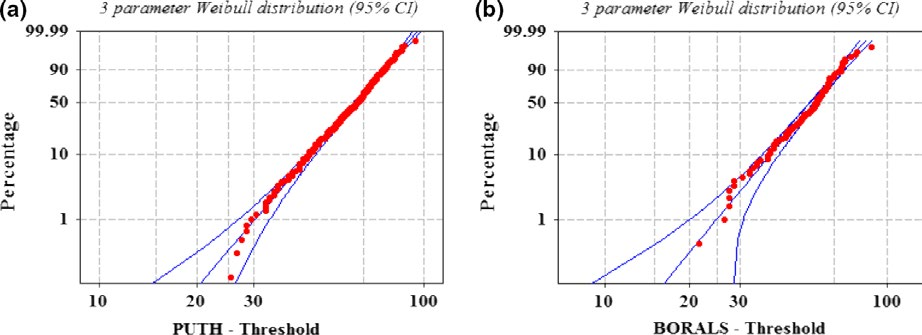
The age distribution of patients overlapped between BORALS and PUTH

Table 2 shows that the male‐to‐female ratio was close. In addition, generally, A–D was <1.5, indicating good fit. The results in Figure 2 and Table 3 show that the age of the patients at PUTH and the age of the overlapping patients between PUTH and the BORALS were consistent with the three‐parameter Weibull distributions. In addition, clearly, the characteristics of the data, namely, the mean, median, and *SD*, were close. Therefore, it was proven that the patient source distribution of the BORALS represented the proportion of all Beijing patients with ALS in each diagnostic hospital. Accordingly, we calculated the number of patients at the Beijing hospitals (see Table 4). A total of 965 patients diagnosed with ALS within the 6‐year period were estimated from 2010 to 2015 in Beijing.

**Table 2:** Sex ratios of patients in PUTH and sex ratios in overlapping patients between PUTH and the BORALS

Data sources	Total	Gender	*N*	Proportion
PUTH	562	Male	346	1.60
		Female	216	
BORALS	165	Male	101	1.58
		Female	64	

**Table 3:** Results of patient age distribution tests for PUTH and in overlapping patients between PUTH and the BORALS

Data sources	Test result			Data characteristics		
	Theoretical distribution	A–D	A–D *p* value	Mean (95% CI)	Median	*SD*
PUTH	Weibull	0.631	0.21	54.06 (52.16, 56.02)	56	11.92
	Beta	0.726	–			
BORALS	Weibull	0.778	0.02	54.27 (52.27, 56.06)	57	11.68
	Beta	0.795	–			

Note

A–D: Anderson–Darling test; CI: confidence interval; *SD*: standard deviation.

**Table 4:** Number of patients diagnosed with ALS in Beijing from 2010 to 2015

Diagnostic Hospitals	Number of patients in BORALS	$\beta_{X}$ (%)	Number of cases in Beijing
PUTH	165	58.30	562
PLAGH	12	4.24	41
PUMCH	53	18.73	181
BJTTH	9	3.19	31
XWHOSP	30	10.60	102
Other	14	4.94	48
Total	283	100	965

### 3.2 Incidence

According to the demographic data, the average annual incidence of ALS in Beijing was 0.8 cases per 100,000 persons (95% CI 0.7, 0.9). The male–female ratio of the third cohort was 1.63:1. In contrast, the incidence of ALS was almost the same multiple as the male–female ratio in men compared with women (0.9 per 100,000 person‐years (95% CI 0.7, 1.1) vs. 0.6 per 100,000 person‐years (95% CI 0.5, 0.8), respectively, *p* < 0.05).

**Figure 3:**
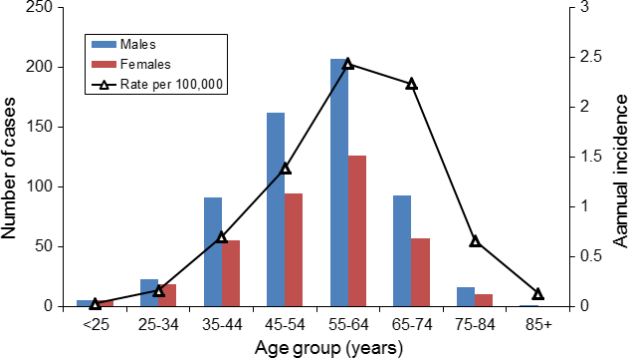
Incidence rates trends of males and females with age

Age at diagnosis ranged from 21 to 88 years; the mean age was 54.11 years, with 81.32% of the patients having been diagnosed between the ages of 40 and 70 years. Men were older than women at the time of diagnosis: 54.76 vs. 53.05 years, respectively. Incidence rates increased with age (*p* < 0.05), peaking at 55–64 years (2.44 per 100,000 persons) and followed by a marked decline (see Table 5 and Figure 3).

**Table 5:** Age distribution and annual incidence of Beijing patients diagnosed with amyotrophic lateral sclerosis, 2010–2015

Age‐group, years	Number of cases (*N*)^a^			Rate per 100,000 (95% CI)^b^
	Males	Females	Total	
<25	5	5	10	0.03 (0.00, 0.08)
25–34	23	19	42	0.16 (0.04, 0.28)
35–44	91	55	146	0.70 (0.42, 0.98)
45–54	162	95	257	1.39 (0.97, 1.81)
55–64	207	126	333	2.44 (1.74, 2.99)
65–74	93	57	150	2.24 (1.36, 3.23)
75–84	16	10	26	0.66 (0.04, 1.28)
85+	1	0	1	0.13 (0.00, 0.73)
Total	598	367	965	0.79 (0.67, 0.91)

a The number of patients with different age‐groups and sex in Beijing was defined according to the third cohort.

b To better reflect the differences between groups, the incidence is accurate to two decimal places.

### 3.3 Clinical features and prognostic analysis

There were 283 patients in the first cohort, and 102 were deceased at the time of the study. We analyzed the site of onset, the time from symptom onset to diagnosis and sex differences among these 283 ALS patients. As a result, 58.3% of the patients were found to have signs of limb disease, with an average age of 51.7 years versus 54.3 years for the patients with bulbar and generalized symptoms at onset (*p* < 0.05). The mean time from symptom onset to diagnosis was 14.8 months. Particularly, bulbar‐onset patients were diagnosed earlier, with a median lag of 9.7 months from the start of symptoms to diagnosis compared with a median lag of 13.2 months for patients with other symptoms (*p* < 0.05). In addition, it was found that the average diagnosis time for women was slightly longer than that of men. (mean lag of 14.9 vs. 13.6 months, respectively; *p* = 0.058).

On the other hand, we also studied the information of the deceased and analyzed the relationship between median survival and gender, age at diagnosis, site of onset, lag from onset to diagnosis, and body mass index (BMI; see Table 6). Due to the relatively small number of ALS deaths in Beijing during 2010–2015, only 102 patients who have died have been collected in our study. Although this data is not much, it is very valuable.

**Table 6:** Median survival based on the patient characteristics

Characteristic	Number of cases	%	Median survival from diagnosis (months)	Log‐rank test *p* value
Total	102	100	49.4	
Gender				
Males	62	60.8	47.8	<0.05
Females	40	39.2	49.2	
Age at diagnosis				
<40 years	11	10.9	59.7	<0.05
40–49 years	24	23.5	51.1	
50–59 years	24	23.5	47.1	
60–69 years	30	29.4	40.9	
≥70 years	13	12.7	40.8	
Site of onset				
Limb	63	61.8	51.3	<0.05
Bulbar	35	34.3	47.6	
Generalized	4	3.9	47.8	
Lag from onset to diagnosis				
≤12 months	59	57.8	47.6	<0.05
>12 months	43	42.2	50.2	
BMI				
<18.5	15	14.7	39.1	<0.05
18.5–24.9	35	34.3	48.4	
≥25	25	24.5	51.1	
Missing	27	26.5	48.2	

The median survival time from diagnosis was 49.4 months. The median survival of males from diagnosis was slightly shorter than that of females (47.8 vs. 49.2 months, respectively). Survival differed based on each patient’s age at the time of diagnosis. Therefore, we separated patients into groups by decade of age, beginning with those younger than 40 and continuing until age 70 or older. The median survival time was 59.7 months in the youngest age‐group, and the value steadily decreased by 51.1, 47.1, 40.9, and 40.8 months, respectively (*p* < 0.05; Table 6), although the median survival between 60–69 and 70 years of age was not significantly different. More than half of the patients with ALS were classified as limb onset, the median survival time of whom was longer than that of patients with bulbar and generalized onset disease (51.3, 47.6, and 47.8 months, respectively, *p* < 0.05). In addition, each patient's survival was related to the length of time between onset and diagnosis, showing a negative correlation. A delay between onset and diagnosis of <12 months was related to a median survival of 47.6 months, as opposed to 50.2 months for those with a delay >12 months (*p* < 0.05). Each patient's BMI at the time of treatment was directly correlated with the length of disease time: In our known data, the median survival increased with increases in BMI values, more importantly, the median survival of patients with BMI values of <18.5 was only two‐thirds that of patients with BMI values of more than 25 (*p* < 0.05, Table 6).

## 4 DISCUSSION

In the present study, we used multiple data sources to estimate the incidence of ALS in Beijing between 2010 and 2015 and conducted a study of the clinical features of ALS. The study provided the following major findings: (a) The incidence of ALS in Beijing was 0.8 cases per 100,000 person‐years. (b) Males are more susceptible to ALS than females, and the incidence of ALS is related to age; and (c) the median survival of patients with ALS is closely related to the age at diagnosis, site of onset, lag from onset to diagnosis, and BMI.

To our knowledge, this is the first specialized study of the incidence of ALS in Beijing. In our study, the estimated incidence of ALS was similar to that of some studies in Hong Kong (0.60/100,000/year; Fong et al., 2016) and Taiwan (0.51/100,000/year; Tsai, Wang, Hwang, Lee, & Lee, 2015). In addition, according to the incidence rates of ALS reported by Marin (Marin et al., 2016), the incidence rates of ALS are lower in the Asian population than in the European and American populations, a result that was also demonstrated in the present study. Recent studies in Europe showed that the incidence of ALS in Europe varies between 1.9 and 2.9/100,000/year (Beghi, Millul, Micheli, Vitelli, & Logroscino, 2007; Logroscino et al., 2005, 2010 ; Sclerosis & Lateral, 2001). Low incidence rates may be related to the genetic background of Chinese patients, even Asians, but this relationship is not yet clear. The incidence rate was consistently higher in males than in females, a finding that was consistent with previous studies (Chio et al., 2013) and might be associated with the fact that males are more likely to be exposed to some environmental risk factors than females or with the possible protective hormonal factors in females. In the next study, we will expand the scope of the study, to include not only the Beijing area, but also in most parts of China, and hope that the results will be more convincing. At the same time, we will study the situation in other parts of Asia to the extent possible. On the one hand, such studies will allow us to look for differences in the epidemiology of ALS across different Asian regions. Such data will also allow us to compare the results with Europe and the United States to look for differences among different regions.

We defined Beijing patients with ALS at the BORALS from 2010 to 2015 as a cohort, different from the report (Chen et al., 2015) of the geographical range of the population and time span, to describe its clinical features. As a result, the mean age at diagnosis in Beijing was found to be about a decade younger than those of European and American patients, an observation that was highly consistent with previous research findings (Chen et al., 2015; Shahrizaila et al., 2016). These facts may suggest that the Chinese population may be exposed to more serious environmental or occupational factors than other populations. In addition, the average life expectancy in China is shorter than that of developed countries, a fact that may relate to this finding. ALS has been found to peak between 55 and 64 years of age, 10 years younger than the ranges found in several European studies (Beghi et al., 2007; Logroscino et al., 2005; Sclerosis & Lateral, 2001), a finding that is consistent with the existence of a genetically susceptible cohort. Several European studies (Beghi et al., 2007; Logroscino et al., 2010) observed more female than male patients in the oldest age‐group, but this was not observed in the present study. The reason for these differences is that the number of cases was small or, due to other reasons, still needs further study.

Disease and patient characteristics, such as gender, age, BMI, or site of disease, are inextricably linked. In the present cohort, there was little difference in the median survival between men and women. We confirmed that many studies suggest that bulbar symptoms predict shorter survival, and the proportion of bulbar disease is generally 1/3 of the total population. In addition, the overall median survival was approximately the same as that of a previous report (Chen et al., 2015) in China, but a slightly shorter survival was observed in the present cohort (56 vs. 49.4 months, respectively); survival was shorter in females than in males. PUTH is the largest ALS center in China, with patients from throughout China. Fewer young patients (who are more capable than old patients of traveling for treatment) and more bulbar‐onset patients, who presumably have worse prognoses, in Beijing contribute to shorter survivals. The proportion of female patients in Beijing is higher than that in the report, which also affects women's survival because women have been observed to have a higher risk of bulbar onset.

Age was the most important predictor of survival in our study. Moreover, younger patients with ALS in our cases were less likely than older patients to be associated with bulbar onset. Younger patients tend to suffer less comorbidity than elderly patients and have better respiratory capacities; thus, younger patients have longer survivals. According to the standards of the WHO, a BMI below 18.5 is defined as underweight, and the risk of disease increases. Indeed, in our study, the median survival of underweight patients with ALS was only half that of normal weight patients, and BMI was also an important prognostic factor.

This paper estimated the incidence of ALS in Beijing. However, there were limitations compared with population‐based registries. The results of the estimation can only be as close as possible to the real situation, and small deviations in the raw data may cause great differences in the estimation results. Nevertheless, reasonable estimations can only be used to quantify incidence under the condition of no patient totals. Although the establishment of population‐based registries and the use of new research methods generate, to some extent, better data and less bias (Logroscino et al., 2008), there are limitations in funding, sample sizes, and the number of years of activity (Jpk et al., 2017). In contrast, estimation methods can mitigate or avoid these limitations.

In summary, this is the first specialized study of the incidence of ALS in Beijing. Future, we focused on the differences in the present study for a better understanding of the current epidemiology of ALS among the Beijing population.

## ETHICAL APPROVAL

Our study was reviewed and approved by the Ethics Committee of the Peking University Third Hospital (PUTH).

## CONFLICT OF INTEREST

The authors report no conflict of interest.
